# Adsorption Behavior and Kinetics of 1,4-Dioxane by Carbon Aerogel

**DOI:** 10.3390/toxics12020145

**Published:** 2024-02-12

**Authors:** Tianyu Lu, Huihui Huang, Guifen Lv, Fei Li, Ren-jie Song, Yuting Cai

**Affiliations:** 1 Key Laboratory of Jiangxi Province for Persistent Pollutants Control and Resources Recycle, Nanchang Hangkong University, Nanchang 330063, China; lty756425125@163.com (T.L.); 2102085700033@stu.nchu.edu.cn (H.H.); yutingcai@163.com (Y.C.); 2 Beijing Construction Engineering Group Environmental Remediation Co., Ltd., Beijing 100015, China; lifei@bceer.com; 3 National Engineering Laboratory for Site Remediation Technologies, Beijing 100015, China

**Keywords:** carbon aerogels, sol–gel method, adsorption, 1,4-dioxane, trichloroethylene

## Abstract

1,4-dioxane is a potential carcinogen in water and is difficult to deal with due to its robust cycloether bond and complete miscibility with water. To remove 1,4-dioxane in an economically viable and environmentally friendly way, a series of carbon aerogels were synthesized as adsorbents for 1,4-dioxane. The experiment results showed that adsorption performances were closely related to the preparation conditions of carbon aerogels, such as the molar ratio, heating rate, pyrolysis temperature and residence time, which were carefully controlled. Scanning electron microscope analysis revealed the presence of a three-dimensional porous network structure in carbon aerogels. Brunauer–Emmett–Teller analysis results demonstrated an increase in specific surface area (673.89 m^2^/g) and total pore volume after carbonization, with an increase in mesoporous porosity and a decrease in microporosity. When considering each variable individually, the highest specific surface area of prepared carbon aerogels was achieved at a pyrolysis temperature of 800 °C, a holding time of 1 h, and a heating rate of 2 °C/min. Under optimal experimental conditions, the adsorption removal of 1,4-dioxane by carbon aerogels exceeded 95%, following quasi-second-order kinetics and Langmuir isothermal adsorption isotherms, indicating that monolayer adsorption on the surface of carbon aerogels occurred. The maximum adsorption capacity obtained was 67.28 mg/g at a temperature of 318 K, which was attributed to the presence of a large proportion of mesopores and abundant micropores simultaneously in carbon aerogels. Furthermore, with the interference of chlorinated solvents such as trichloroethylene (TCE), the removal efficiency of 1,4-dioxane had no obvious inhibition effect. Regeneration experiments showed that after five continuous cycles, the carbon aerogels still kept a comparable adsorption capacity, which illustrates its potential application in 1,4-dioxane-polluted water purification.

## 1. Introduction

1,4-dioxane (1,4-D) has been identified as an impurity in numerous personal care products and cosmetics, as well as an undesired by-product in various industrial processes including antifreeze production, surfactant manufacturing, and paint formulation. The specific physical properties of 1,4-dioxane are shown in Table 1. The historical utilization, improper storage practices, and continuous discharge of 1,4-D have led to extensive contamination in drinking water sources, surface waters, and groundwater [1]. Once released into the environment, 1,4-D persists and exhibits high mobility due to its robust cyclic ether linkage and complete miscibility with water [2,3,4]. Toxicological studies have indicated that exposure to 1,4-D can cause health harms such as nasal cavity carcinomas and liver and gall bladder carcinomas [5]. Therefore, it has been classified as a Group 2B probable human carcinogen by the International Agency for Research on Cancer (IARC) [6]. Data show that approximately 21% of the drinking water supplies in the United States are contaminated with 1,4-D [7]. Counties including Japan, South Korea, and Canada have proposed a guideline for the concentration of 1,4-D in drinking water of 50 μg L^−1^. Though 1,4-D is not federally regulated in the US, several states have established notification levels and guidelines. The acceptable concentrations of 1,4-D are low, typically in the range of (sub-) parts per billion (ppb), e.g., New York State set up a level of 1 µg L^−1^, and several other states have established water criteria with the concentration ranging from 0.3 to 7.2 µg L^−1^ [6]. The prevalence, persistency, and toxicity of this emerging pollutant have prompted increasing attention, highlighting the necessity for cost-effective treatments to mitigate its exposure risk [8].

Furthermore, its low Henry’s law constant, low Kow and low Koc limit the effective re-mediation of 1,4-D via conventional technologies. For instance, air stripping is not efficient in treating 1,4-D due to its low volatility and high hydrophilicity [9]. Advanced oxidation processes (AOPs) [10] are effective alternatives to treat 1,4-D, including electrochemical methods, photo-catalysis [11], Fenton reaction methods [12], etc. The AOPs exhibit high removal efficiency and a short reaction time. However, these methods necessitate substantial energy consumption and incur significant costs, often resulting in secondary environmental pollution. Additionally, the degradation products generated by AOPs typically consist of low-molecular-weight organic compounds that require further treatment. In summary, there is a demand for economically viable and environmentally friendly approaches to 1,4-D treatment.

Adsorption has been a conventional and successful method for the removal of contaminants due to its simplicity, cost-effectiveness, and ease of operation. As shown in Table 2, some porous materials have been studied for the adsorption of 1,4-D. Among these adsorbents, some showed low adsorption capacity towards 1,4-D or entail a complex preparation process and have high costs. Pollutants such as persistent organics [13,14], dyes [15] and inorganic metals [16,17] can be adsorbed efficiently via granular activated carbons (GAC). Unlike other contaminants, 1,4-D cannot be removed efficiently by GAC because of its special characteristics including low solubility, high water solubility and other characteristics [18,19]. Nevertheless, studies have demonstrated the effective removal of 1,4-D by Ambersorb 560 (A560) [20], a carbonaceous adsorbent synthesized by the Dow Chemical Company, Midland, USA. A560 possesses a high surface area and porosity that facilitate specific adsorption of 1,4-D. Additionally, it can be regenerated using low-pressure steam. Nonetheless, A560 is relatively expensive due to its complex manufacturing process. Therefore, the development of alternative adsorbents is desirable to meet the increasing demand for the sustainable and economical management of dioxane-impacted water worldwide.

Aerogels are porous materials with many unique characteristics such as low bulk density, large surface area, low thermal conductivity, and porosity > 95%. Aerogels can be made of SiO_2_, graphene, MoS_2_ and so on. Pekala et al. [21] first reported the synthesis of porous resorcinol (R) and formaldehyde (F) aerogels, and after undergoing carbonization in an inert atmosphere, the RF aerogel underwent a transformation into a carbon aerogel (CA) [22]. CAs exhibit the characteristics of being porous, lightweight, and possessing a large specific surface area [23]. They have the ability to adsorb various pollutants, including inorganic metal ions [24,25,26] and organic pollutants [27]. For instance, CAs have shown excellent adsorption capacity on pesticides [28], 4-nitrophenol [29] and dyes [30]. In particular, CAs have shown a promising adsorption performance on some pollutants that were difficult to be adsorbed on GAC, especially for cyclic substances such as benzene rings or antibiotics with large molecular weights [31].

**Table 2:** Comparison of adsorption properties of other materials for 1,4-dioxane.

Adsorbent	Initial Solute Concentration	Dosage	Q_max_ (mg-1,4-D g-Adsorbent^−1^)	Cycles (Recovery Rate)	Equilibrium Time	Reference
Norit 1240 GAC	100 mg/L	1 g	~38	_	72 h	[19]
Ambersorb 560	80 mg/L	_	~40	_	_	[32]
Titanium Silicate-1	298 K, 500 mg/L	100 mg	85.17	3 (~100%)	2 min	[33]
Sawdust GAC	296 K	_	0.41	_	16–24 h	[18]
ZSM-5 zeolite	303 K, 100 mg/L	_	22.44 to 107.36	_	1–8 h	[34]
CA125-800-1-2°	308 K, 20 mg/L	20 mg	67.28	5 (~100%)	1 h	[This work]

However, little is known regarding the effectiveness of CAs for 1,4-D adsorption and the associated underlying mechanisms. In this study, through the sol–gel method, resorcinol and formaldehyde are utilized as precursors, while cetyltrimethylammonium bromide (CTAB) is introduced as a surfactant and catalyst. The continuous cross-linking and room temperature drying of resorcinol and formaldehyde result in the formation of a phenolic resin-based aerogel during the reaction process. Carbonization at high temperatures under an inert atmosphere is employed to prepare CAs. This study not only investigates the impact of different carbonization procedures on the adsorption performance and structural characteristics of CAs, comparing their adsorption rates with A560 and activated carbon for 1,4-D removal, but also analyzes the kinetic and thermodynamic models governing CAs’ adsorption process. Additionally, this research explores the underlying mechanism behind 1,4-D removal by CAs through comprehensive surface characterization. In addition, as stable and renewable adsorbents, CAs elicit potential values and unparalleled interest in the fields of water treatment and remediation due to their efficient and economic benefits.

## 2. Materials and Methods

### 2.1. Chemicals and Materials

Resorcinol (C_6_H_6_O_2_, 99%) and formaldehyde (CH_2_O, 36~38%) were provided by TCL Chemical Products Co., Ltd. (Guangzhou, China), and cetyl trimethyl ammonium bromide (CTAB) was purchased from Adamax Reagent Co., Ltd. (Shanghai, China). 1,4-dioxane (C_4_H_8_O_2_,99%) was provided by Anneji Chemical Technology Co., Ltd. (Shanghai China), and deionized water (resistivity < 18.2 MΩ, Millipore system) was used. Ambersorb 560 (A560) was purchased from the Dow Chemical Company, and GAC (200 mesh) was purchased from Thain Chemical Technology Co., Ltd. (Shanghai, China).

### 2.2. CAs Preparation

**Figure 1:**
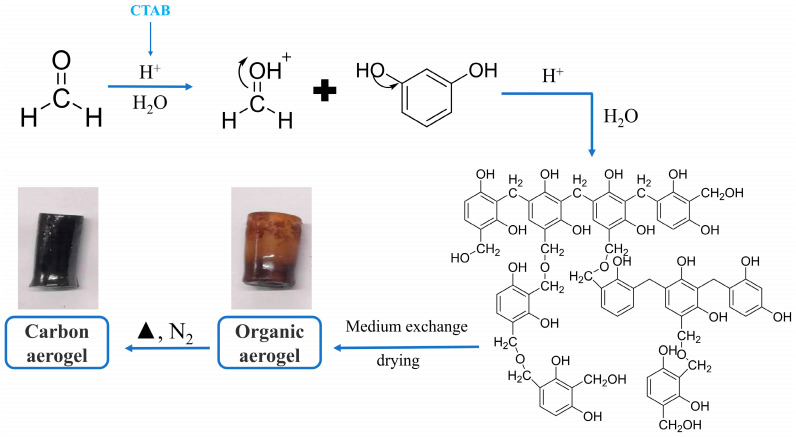
Preparation process of CAs.

The preparation of CAs was based on the sol–gel polymerization method [35]. Briefly, a predetermined amount of resorcinol (R), 37% formaldehyde solution, cetyltrimethylammonium bromide (CTAB) and deionized water were added into a glass beaker, while stirring well at room temperature for 30 min, and then transferred into a glass vial. The vial was sealed and placed into an oil bath pot at 85 °C for 5 days, and the obtained organic aerogel was cured at room temperature for 2 days, followed by 60 °C for 24 h and then 105 °C for 3 h in an oven. Finally, the dried organic aerogel was heated to the point of carbonization with a set heating rate and residence time in a tube furnace. The molar ratios of R/CTAB were from 50 to 500, the carbonization temperatures ranged between 700 °C and 800 °C, the heating rates were 2 °C/min and 5 °C/min, respectively, and the residence time was in the range of 1 h to 2 h, respectively. They were denoted as CAa-b-c-d, where a represented the molar ratio R/CTAB, b represented the carbonization temperature, c represented the residence time, and d represented the heating rates. The specific preparation process is shown in Figure 1.

### 2.3. Characterization

The specific surface area and pore size of the CAs were measured using a specific surface area analyzer (BELSORP-max, MicrotracBEL Japan, Inc., Osaka, Japan). The surface morphology was investigated via scanning electron microscopy (SEM, S-3400N, Hitachi, Japan). Powder X-ray diffraction (XRD) pattern was performed on a D8-Advance-A25 diffractometer with Cu Kα radiation (λ = 1.5418 Å) at 40 kV and 200 mA and a step size of 0.02° in the 5°–90° 2θ range at room temperature (25 °C). The pH of the point zero charge was measured via a potentiometric titrimeter (ZDJ-4A, Leici, Shanghai Instrument Electrical Scientific Instrument Co., Ltd., Shanghai, China).

### 2.4. Adsorption Experiments

Adsorbents screening experiments were evaluated in batch tests prepared in 25 mL glass flasks containing 60 mg of adsorbents (CAs, A560, GAC) and 10 mL 1,4-D with initial 20 mg/L concentrations, and the temperature was measured at 298 K. The mixture was shaken in a thermostatic shaker at 25 °C and 150 rpm for 12 h at room temperature to achieve equilibrium. The supernatant was filtered and analyzed for the concentrations of 1,4-D determined via gas chromatography with flame ionization detection (GC-FID). All experiments were conducted in 3 replicates.

For kinetics experiments, the dosage of CAs was administered at 20 mg and with varying initial 1,4-D concentrations ranging from 20 to 160 mg/L. Moreover, 0.5 mL of supernatant was removed using a syringe and filtered before storage for analysis. Two kinetic models, pseudo-first-order and pseudo-second-order equations which included all steps of the adsorption such as external film diffusion, adsorption, and internal particle diffusion, were used to analyze the adsorption processes. The formula is shown in Equations (1) and (2).

(1) $$\ln\left(Q_{e}-Q_{t}\right)=lnQ_{e}-k_{1}t$$

(2) $$\frac{t}{Q_{t}}=\frac{1}{k_{2}{Q_{e}}^{2}}+\frac{1}{Q_{e}}t$$

For adsorption isotherms, batch experiments were prepared similarly as described above, but with varying initial 1,4-D concentrations ranging from 20 to 480 mg/L, while the experimental temperature varied between 298 K and 308 K. The maximum sorption capability (Q_m_) and other parameters were computed by fitting with classic isotherm models (e.g., Langmuir and Freundlich) [36].

The Langmuir model is shown in Equation (3):

(3) $$\frac{C_{e}}{Q_{e}}=\frac{1}{Q_{m}K_{l}}+\frac{C_{e}}{Q_{m}}$$

The Freundlich model is shown in Equation (4):

(4) $$\log Q_{e}=\log K_{f}+\frac{1}{n}\log C_{e}$$

where Q_e_ (mg/g) and C_e_ (mg/L) were the amounts of adsorbed 1,4-D per unit mass of adsorbent at equilibrium. Q_m_ (mg/g) was the maximum amount of 1,4-D per unit mass of the adsorbent to form a complete monolayer on the surface. K_l_ (L/mg) was a constant related to the affinity of the binding sites on the adsorbent. K_f_ (L/mg) and 1/n were the Freundlich model constants, indicating the capacity and intensity of adsorption, respectively.

Adsorption thermodynamics enables the investigation of various factors, including temperature, on the adsorption process. In terms of temperature’s impact, a set of thermodynamic parameters can be derived to substantiate its influence on 1,4-D adsorption via CAs. These parameters primarily encompass Gibbs free energy (∆G), adsorption enthalpy change (∆H), and adsorption entropy change (∆S), which can be computed using Equations (5) and (6):

(5) ∆G=−RTlnK

(6) $$lnK=\frac{∆S}{R}-\frac{∆H}{RT}$$

In the given formula, R represents the universal gas constant with a value of 8.314 (J/mol/K). T denotes the absolute Kelvin temperature (K) of the adsorption solution, while K signifies the dimensionless thermodynamic equilibrium constant. The K_L_ (L/mg) value from Langmuir’s thermodynamic curve is multiplied by 10^6^ to obtain its corresponding numerical value. Herein, consider 1/T the *x*-axis and lnK the *y*-axis. Furthermore, ΔH and ΔS can be determined by calculating the slope and intercept of an equation that relates lnK to 1/T.

### 2.5. Analytical Methods

1,4-D concentration was detected via GC-FID (Agilent 7890A, Thermo, Waltham, MA, USA) coupled with a Supelco SLB™-5 ms fused silica capillary column (30 m length × 0.2 mm ID × 0.25 μm film). The direct injection volume of the filtered aqueous sample was 1 µL. Nitrogen was used as the carrier gas with a constant flow rate of 6.0 mL/min. The inlet temperature was set at 250 °C, and the samples were split at the ratio of 2:1 by the split flow of 12 mL/min. The oven temperature started from 110 °C for 1 min, then ramped up to 180 °C at the rate of 15 °C/min, and was held for 6 min. The detector temperature was maintained at 250 °C.

### 2.6. Regeneration Experiment

1,4-D-laden CA was regenerated via extraction with ethanol and then by heating the used CAs in an oven at 110 °C for 12 h. Adsorption experiments were repeated by using the same CA in consecutive cycles. After each desorption cycle, the CA was reused in a new adsorption process. The adsorption–desorption test of the adsorbent was repeated for 5 consecutive cycles. Data for the adsorption and regeneration experiments were calculated as the average value of the three replicates.

## 3. Results

### 3.1. The Investigation of Adsorption Properties

**Figure 2:**
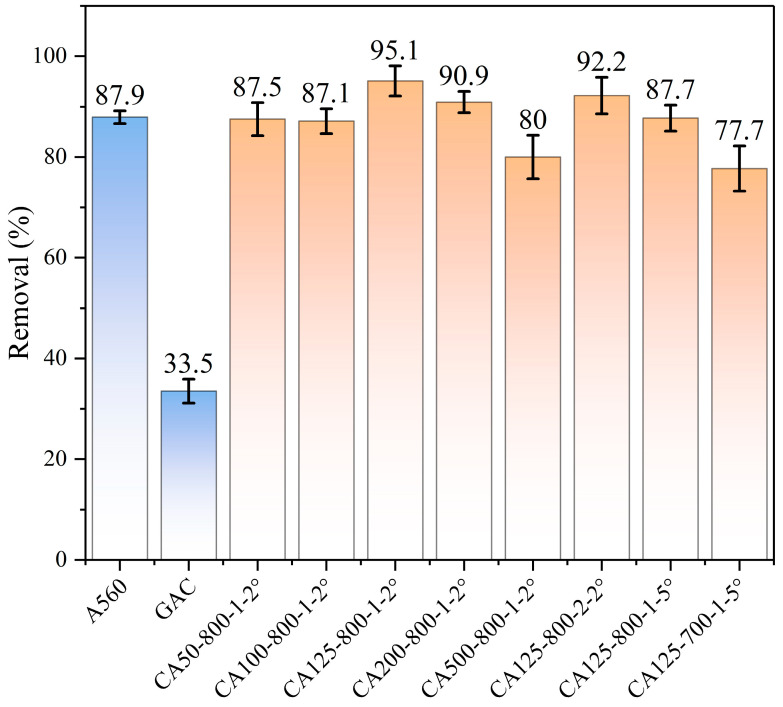
Removal effects of CAs, A560, and GAC on 1,4-D.

A series of experiments for CA preparation were performed to optimize the 1,4-D removals using CAs. The CAs’ synthetic conditions included the R/CTAB ratio, pyrolysis temperature, residence time and heating rate. As Figure 2 showed, the concentration of 1,4-D is 20 mg/L with an addition amount of CAs at 60 mg and an adsorption volume of 10 mL at room temperature (298 K). The preparation conditions affected the 1,4-D removal greatly. A560 and GAC were used as controls, and the removal amounts were 87.89% and 33.47%, respectively. All CAs showed much higher removal than GAC. The R/CTAB ratio varied from 50 to 200, and the highest 1,4-D removal was obtained at R/CTAB = 125, which was 95.08%. When the pyrolysis temperature increased from 700 °C to 800 °C, the 1,4-D removal increased first and then decreased. The optimum temperature was 800 °C. Similarly, there was some variation from 1 h to 2 h. The optimum residence time and heating rate were 1 h and 2 °C/min, respectively. Therefore, the optimum CA candidate was CA125-800-1-2°. The removal of 1,4-D reached a high percentage of 95.08%, which was 1.1 times higher than that of A560 and 2.8 times higher than that of GAC, respectively. For the sake of convenience for later descriptions, the following discussion uses CA125 referring to CA125-800-1-2°.

### 3.2. The Effect of CAs’ Porous Structure

**Figure 3:**
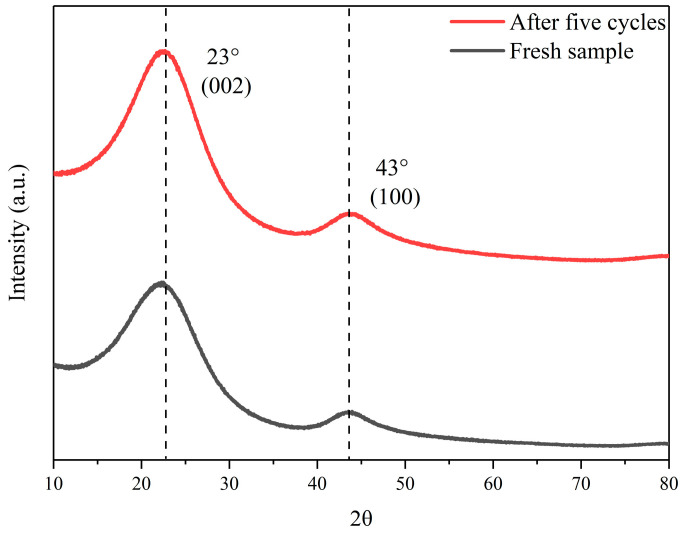
XRD spectra of CA125-800-1-2° before and after the reuse experiments.

The adsorption performance is strongly related to the micro-structural natures of the adsorbent. The crystal structure of CA125 was analyzed via XRD as shown in Figure 3. The diffraction peaks at 23° and 43° correspond to the layered ordered stacked (002) surfaces and the ordered hexagonal carbon structure (100) surfaces. It was shown that CAs are graphite-like microcrystalline carbon materials. The structures resembling graphite exhibit an extended, multi-layered arrangement of unidirectional structural units, and the adsorption process of CAs is enhanced under these conditions [37].

**Figure 4:**
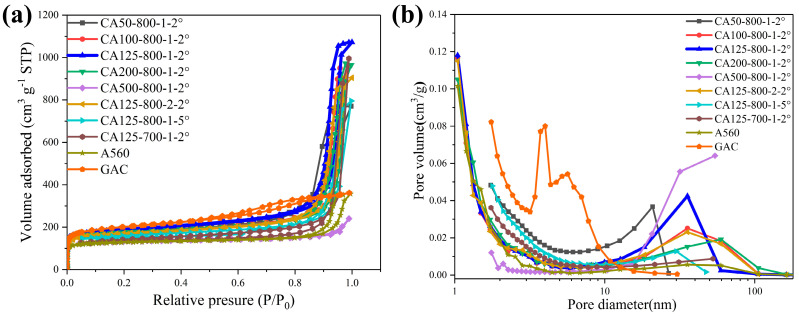
Characterizations of CAs, A560 and GAC: nitrogen adsorption–desorption isotherms (**a**) and pore size distribution (**b**).

The specific area (SA) and pore size distribution of the adsorbents showed significance for 1,4-D adsorption. It is believed that not only the surface area but also the pore size distribution determines 1,4-D adsorption. Figure 4a shows a BET surface area isotherm, and the pore structure parameters are listed in detail in Table 3. According to IUPAC, the N_2_ adsorption–desorption isotherm was the type IV isotherm with the type H3 loop, indicating the formation of developed mesoporous and microporous structures. Among the adsorbents, GAC exhibited the lowest adsorption capacity despite having the largest SA, demonstrating that the surface area is not the key factor for determining the adsorption performance.

In order to further distinguish the pore size distribution of the carbons, their cumulative pore volume curves are analyzed (Figure 4b). CAs showed a wide pore size distribution which can be attributed to the interval gap between chains of interconnected particles. Furthermore, CA125-800-1-2° showed a sharp mesopore distribution centered at 30 nm.

As can be seen from Table 3, though the total SA of CA is not the largest, it showed the highest S_meso_ and V_meso_. S_meso_ of A560 is also lower than CAs. The order is in accordance with the 1,4-D removal via adsorbents, suggesting the mesopores contribution to 1,4-D adsorption. The 1,4-D molecule possesses two O atoms. It is a polar nonionic compound, according to the literature reported [38], and hydrogen bonds can be formed with H_2_O molecules. The diameter of the 1,4-D molecule and H_2_O molecule are ca. 0.5 nm and 0.4 nm, respectively, and hydrogen bonds may be formed between few 1,4-D and H_2_O molecules. The size of the molecule group is a few nanometers long, so mesopores are fit for 1,4-D molecule transfer. The mesopores are 1,4-D storage and diffusion channels. The existence of the mesopore should be a crucial factor for 11,4-D adsorption.

The observed dependence of 1,4-D uptake on pore size is consistent with previous reports. Huang et al. [39] reported a polystyrene-based hierarchical porous carbon (PS-HPC) for supercapacitors. In their view, one of the important interactions for ion transport is the ion–wall pairs. It depends on the ratio of the pore diameter to the ion diameter. When the ratio is larger than 20, the ions transfer into the pores freely, avoiding collisions between the ion and wall. Therefore, with a larger mesopores proportion, CAs showed a higher 1,4-D uptake amount.

**Figure 5:**
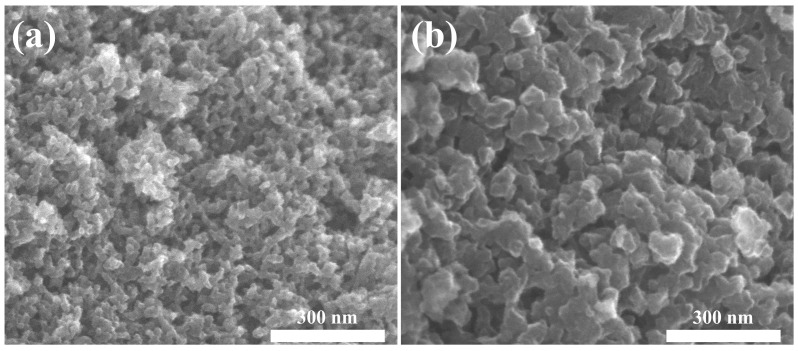
SEM of CA125 (**a**) and A560 (**b**).

According to the SEM image, as depicted in Figure 5, the CA nanoparticles exhibit a size of approximately 30 nm and display an interconnected three-dimensional network structure. This architecture facilitates the transportation and adsorption of 1,4-D. Importantly, the surface of CAs is characterized by abundant pores and defect structures, leading to an increased number of adsorption sites and an enhanced pollutant adsorption capacity. In contrast, although A560 shares a similar structure with CA, it exhibits significantly fewer surface pores and defects. Therefore, based on the aforementioned characterization analysis, it can be concluded that a high mesoporous porosity and a rich pore structure exert a profound influence on the adsorption behavior towards 1,4-D.

**Figure 6:**
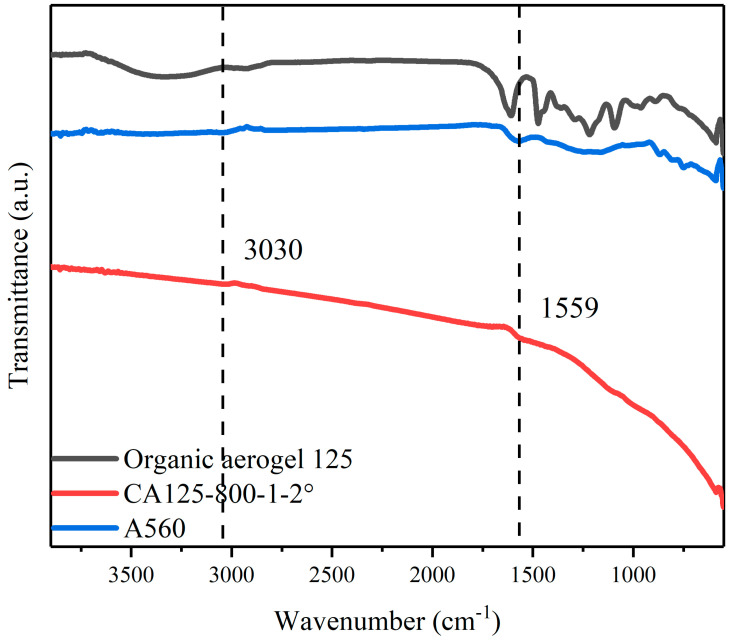
FTIR of organic aerogel 125, CA125-800-1-2° and A560.

The changes in surface functional groups before and after the carbonization of CAs were investigated using Fourier infrared spectroscopy. The infrared spectra of the aerogel (R/CTAB = 125) before and after carbonization, as shown in Figure 6, reveal a rich variety of surface functional groups in the pre-carbonized aerogel. For instance, the absorption peak at 3342 cm^−1^ corresponds to the stretching vibration of –OH, which is broadened due to hydrogen bonding. The absorption at 2927 cm^−1^ is attributed to the asymmetric stretching vibration of C–H. The peak at 1605 cm^−1^ indicates the characteristic C=C stretching vibration in an asymmetric ring, while peaks at 1473 cm^−1^ represent methylene bonds, and those at 1094 cm^−1^ and 1237 cm^−1^ correspond to methylene ether bonds. Additionally, peaks observed at 1358 cm^−1^ and 1290 cm^−1^ are associated with C–N stretching vibrations. A weak peak detected at 961 cm^−1^ can be assigned to either C–N or C–O stretching vibrations. Furthermore, absorptions within the range of 900~650 cm^−1^ indicate out-of-plane deformation vibrations for both C–H or N–H bonds. However, following the carbonization of the aerogel, most surface functional groups disappear, except for the presence of a tensile vibration peak corresponding to a C–H bond at around 3030 cm^−1^ and a benzene unsaturated bond tensile vibration peak observed near 1559 cm^−1^. These results demonstrate that carbonized CAs primarily consist of benzene rings, with other remaining functional groups being decomposed and removed during high temperature treatment.

In summary, CAs belong to a graphite-like amorphous carbon structure, exhibiting a hexagonal carbon framework composed of phenyl rings. They possess a large specific surface area and abundant micro-mesoporous characteristics, rendering them highly effective adsorbents.

### 3.3. 1,4-D Adsorption at Different pH

**Figure 7:**
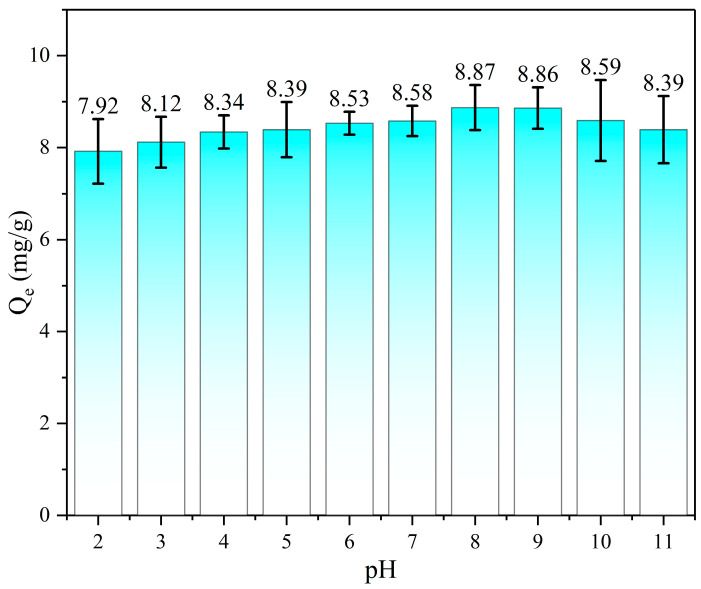
Influence of different pH on adsorption capacity.

The effect of the initial solution pH on 1,4-D removal was investigated by varying the solution pH from 2 to 11, and the results are presented in Figure 7. As the pH value increased, the removal did not show obvious differences. The reason is 1,4-D is in the ion form at the entire pH range since the pka of 1,4-D is 2.1. Further, the zero charge point of CAs is 7.1 [40]. Therefore, a pH without adjustment (pH = 6.6) is recommended due to the negligible effects of electrostatic attraction to 1,4-D adsorption by CAs.

### 3.4. Adsorption Kinetics and Isotherm

**Figure 8:**
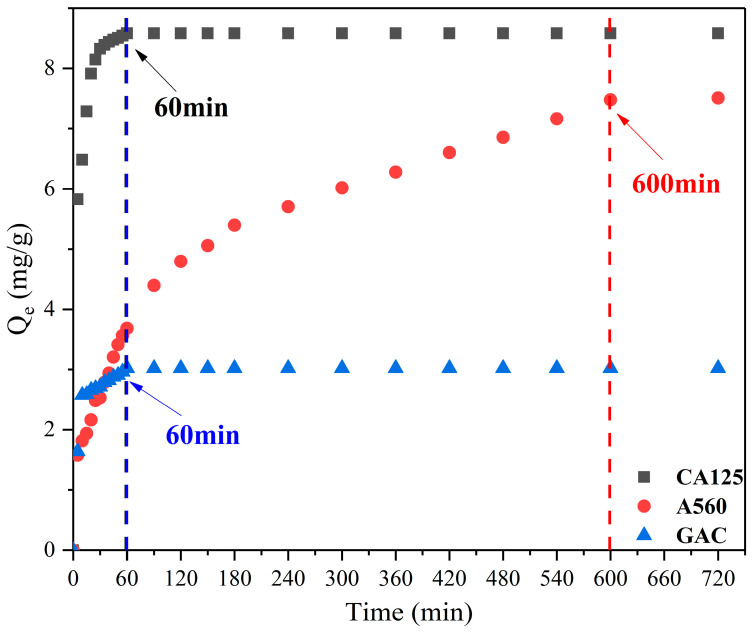
The effect of contact time on the adsorption capacity of 1,4-D over CA125-800-1-2°, A560 and GAC.

The adsorption of CA125 toward 1,4-D with time is showed in Figure 8. The adsorption performance followed the order of GAC < A560 < CA125. In comparison to A560 and GAC, CA125 exhibited not only a significant increase in adsorption capacity but also a considerably shorter equilibration time. Specifically, the adsorption capacity of CA125 was nearly three times that of GAC, while its equilibration time was only one-sixth of that of A560. Within 30 min, the removal efficiency for 1,4-D reached 66.82%, and complete adsorption equilibrium was achieved within 60 min. Conversely, although A560 demonstrated a higher removal capacity than GAC, it required an extended equilibration time of up to 12 h; on the other hand, GAC showed a shorter equilibration time (60 min) but a relatively lower adsorption capacity.

**Figure 9:**
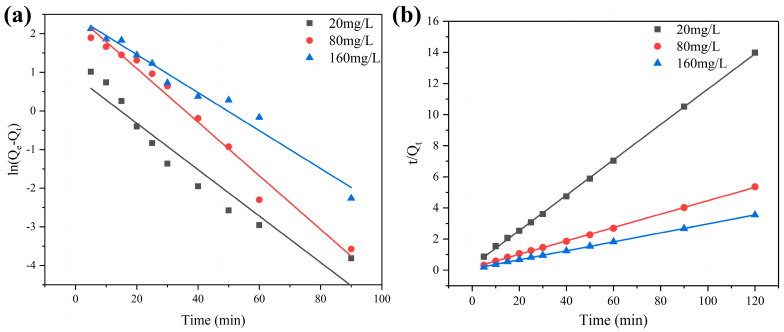
Pseudo-first-order (**a**) and pseudo-second-order (**b**) kinetic models for adsorption of 1,4-D on CA125.

Figure 9a,b shows the pseudo-first-order and pseudo-second-order kinetic models of adsorption tests on CA125 based on different concentrations of 1,4-D. The related parameters are shown in Table 4. As the results show, the R^2^ values of pseudo-second-order models were higher than those of the pseudo-first-order model, suggesting that the pseudo-second-order model can preferably describe the adsorption process. In the pseudo-second-order model, the rate-limiting steps in the surface adsorption involves chemisorption, which means the adsorption process is a physicochemical interaction between 1,4-D and the CA125 surface, where 1,4-D makes contacts with the surface of the CA and binds strongly to the surface.

**Figure 10:**
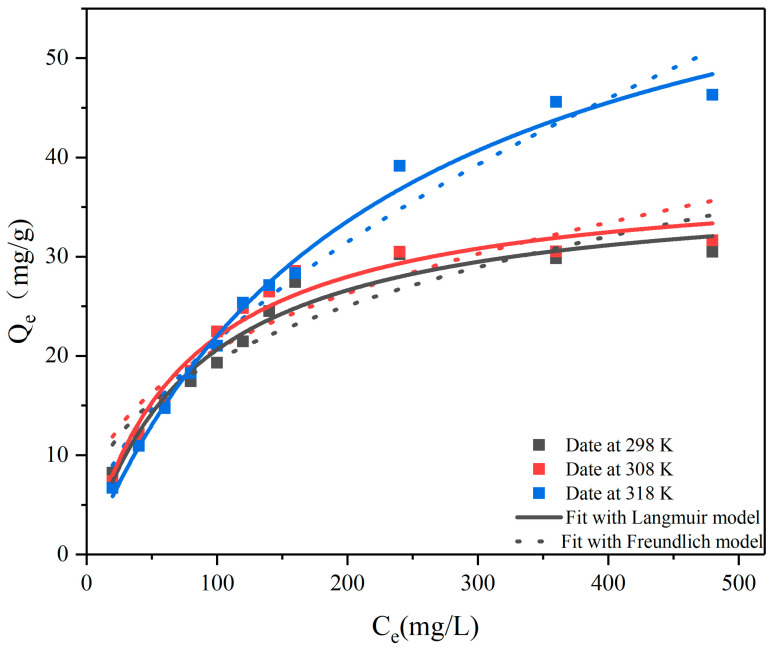
The adsorption isotherm model for 1,4-D on CA125-800-1-2°.

Langmuir and Freundlich adsorption isotherms (Figure 10) were obtained at different temperatures via batch experiments. As the temperature increased, the adsorption capacity increased, indicating that the adsorption of 1,4-D on CA125 is an endothermic process. Furthermore, as the concentration of the initial aqueous solution of 1,4-Dincreased, the adsorption capacity of CA125 increased. The parameters shown in Table 5. The coefficients (R^2^) value of the Langmuir model is higher than that of the Freundlich model, indicating that the Langmuir model is more consistent with the experimental data, which showed that the adsorption 1,4-D on CA125 is a monolayer adsorption process, and the maximum adsorption capacity of 1,4-D was 67.28 mg/g under 318 K, which was very close to the experimental value.

The fitting equation for LnK, obtained through thermodynamic curve fitting can be expressed as LnK = 6339.6 (1/T) − 10.343. Furthermore, the correlation coefficient is R^2^ = 0.93. The calculated results of the relevant parameters for thermodynamic analysis are presented in Table 6. All values of Gibbs free energy ΔG are negative, indicating the spontaneous adsorption of 1,4-D by CAs. Furthermore, the magnitude of Gibbs free energy increases with rising temperatures in the adsorption system solution, suggesting a deteriorating adsorption performance of CAs towards 1,4-D [41]. The negative value of ΔS implies that the adsorption process exhibits a decrease in entropy when CAs are used as adsorbents for 1,4-D. This suggests that an enthalpy change drives the adsorption process [42]. In addition, ΔH is negative within the studied adsorption system, signifying an exothermic nature during the adsorption process of 1,4-D. Moreover, it can be concluded that increasing the temperature inhibits this adsorptive behavior based on consistent findings from Gibbs free energy analysis.

### 3.5. Competitive Adsorption of 1,4-D and Trichloroethylene

**Figure 11:**
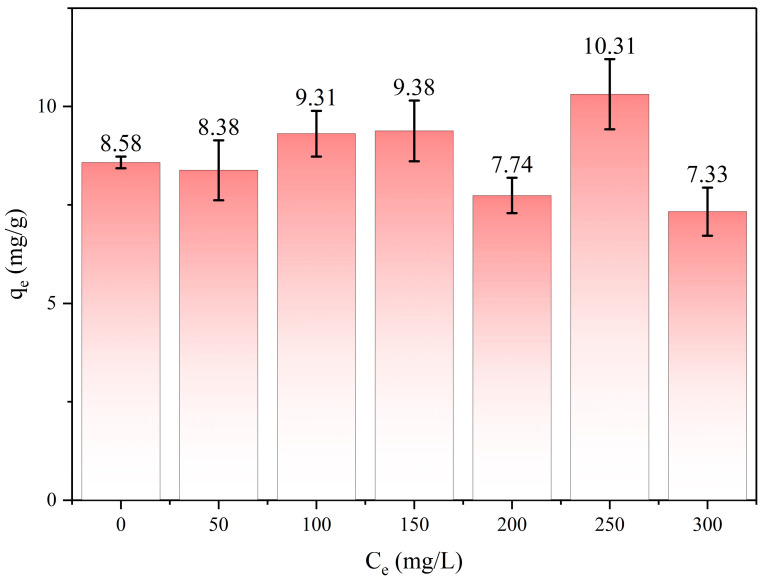
The effects of different concentrations of trichloroethylene on 1,4-D adsorption. C_1,4-D_ = 20 mg/L, T = 25 °C, t = 2 h. Concentrations of TCE are 50, 100, 150, 200, 250, 300 mg/L, respectively.

1,4-D has been predominantly used as a stabilizer for chlorinated solvents such as trichloroethylene (TCE). Hence, the adsorption capacity of CA125 toward 1,4-D was explored with the presence of TCE co-existing in wastewater. As shown in Figure 11, the TCE concentrations were tested between 50 mg/L and 300 mg/L. Compared with the 1,4-D adsorption without TCE, the adsorption capacity was not affected by TCE. Since 1,4-D molecules are neutral amphiphilic, possessing both alkyl and oxygen groups, both hydrophobic and hydrophilic interactions are found to have an important contribution to its adsorption on various adsorbates. The total acidity for the samples of CAs is greater than the total basicity. This is due to the oxidation process undergone by the polymeric gel in the drying process, which induces the formation of more acidic oxygenated functional groups including the carboxylic, lactic, and phenolic groups [43]. The presence of these groups, although in a small proportion, induced the formation of delocalized π electrons and an H bond with 1,4-D molecules. However, TCE is hydrophobic and has a weak interaction with CA, so the adsorption capacity is quite low.

### 3.6. Regeneration of CAs

**Figure 12:**
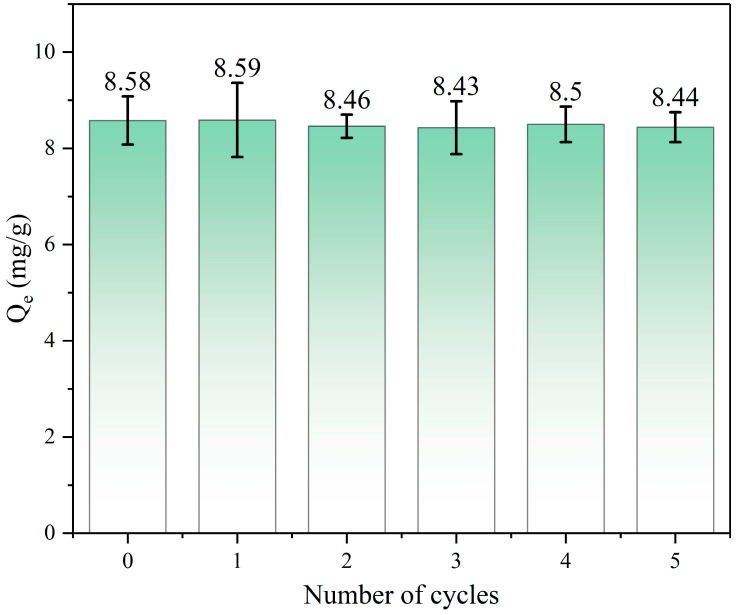
Removal quantity of CA125 after regeneration in five cycles. Cycle 1 represents the original CA125. (C_0_ = 20 mg/L, T = 25 °C, t = 2 h and m = 20 mg).

The regeneration performance of adsorbents is one of the important considerations for practical applications. Figure 12 shows the five consecutive adsorption/desorption cycles to remove 1,4-D. The adsorption removal amount was 8.58 mg/g in the first cycle. The adsorption efficacy of the material remains essentially unaltered upon multiple reuses in comparison to its initial adsorption rate. Moreover, when combined with XRD analysis, the configuration of CAs remained essentially unaltered even after repeated utilization. These results showed that the marked retention of the sorption capability is ascribed to the stable structure of CAs. When advantages feature in the CAs’ adsorbent capacity in repeated cycles of 1,4-D, loading and elution are taken into consideration. These results illustrate the facile and high regeneration capability of CAs as a promising adsorbent for 1,4-D.

### 3.7. The Impact of the Adsorbent Quantity

To compare the adsorption effects of CAs, A560 and GAC adsorbents with different weights on 1,4-D, adsorbents weighing 20 mg, 40 mg, and 60 mg were selected to adsorb a solution containing 10 mL of a 20 mg/L concentration of 1,4-D. The obtained results are presented in Table 7. According to the experimental findings, under ambient conditions, the adsorption efficiency of 0.06g CA125-800-1-2° for a 10 mL solution containing 20 mg/L of 1,4-D can attain an impressive rate of 95%.

### 3.8. The Investigation of the Mechanism

**Figure 13:**
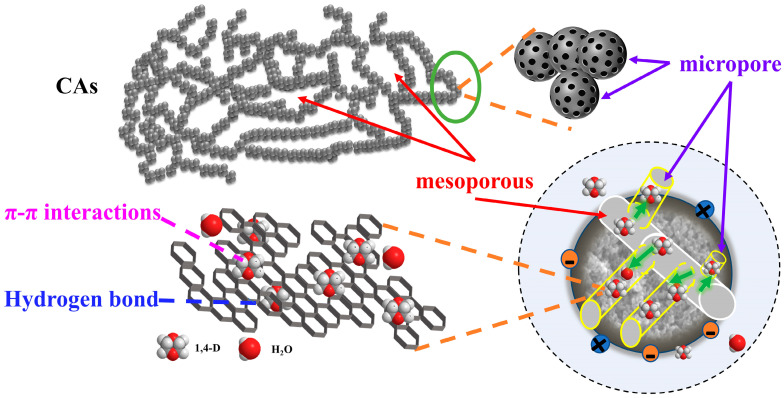
Adsorption mechanism diagram of CAs.

The interactions between the adsorbent and adsorbate involve various forces, such as van der Waals forces, electrostatic attractions, pore filling, π-π electron donor–acceptor interactions, hydrogen bonding, coordination adsorption, and hydrophobic interactions [44,45]. The adsorption mechanism of 1,4-D caused by CAs is depicted in Figure 13. A specific description for this process is as follows:

(1) Pore filling: according to the conclusions drawn from BET analysis (Table 3), the adsorption rates of 1,4-D by the three adsorbents (Figure 2) and the nitrogen absorption and desorption curve (Figure 4), it can be concluded that the adsorption form of CAs on the refractory organic pollutants is mainly physical adsorption, and the adsorption method is mainly pore filling, and the specific surface area and pore volume of carbon materials are larger. This enables a greater the adsorption capacity of 1,4-D.
(2) Van der Waals force action: according to the adsorption dynamic equilibrium curve of 1,4-D, it can be seen that the adsorption of 1,4-D by CAs is mainly physical adsorption. Since the main force in the physical adsorption process is the van der Waals force, it can be inferred that the adsorption of 1,4-D by CAs has van der Waals forces.
(3) Electrostatic attraction mechanism: the surface of CAs is usually negatively charged, which makes it easier for CAs to electrostatically adsorb positively charged organic compounds to form a stable adsorption interface. From a microscopic point of view, since 1,4-D is a polar solution, and polar molecules hydrogen and oxygen have different abilities to capture electrons, the shared electron pairs of the two will favor oxygen, resulting in a positive charge of oxygen, a negative charge of hydrogen, and a prominent oxygen atom. Therefore, 1,4-D will be absorbed to the surface of CAs due to the electrostatic attraction of the oxygen atom.
(4) Electron donor/acceptor effect: according to infrared spectrum analysis, CAs contain a benzene ring as an electron acceptor and the -O- in the ether bond of 1,4-D as an electron donor. The two can enhance the adsorption capacity between the adsorbent and adsorbent through the electron donor/acceptor complex effect.

## 4. Conclusions

The adsorption behavior of a series of CAs adsorbents and their interactions with 1,4-D were investigated. It was observed that CAs exhibited excellent adsorption capacity for 1,4-D in aqueous solutions. Notably, CA125-800-1-2° possessed a large specific surface area and abundant micro-mesoporous structure, resulting in a remarkable removal efficiency (~95%) for 1,4-D in water. A kinetic simulation revealed the maximum unit adsorption capacities at 298 K and 318 K to be 37.55 mg/g and 67.28 mg/g, respectively. Furthermore, the high adsorption rate coupled with the low desorption tendency indicated that the quasi-second-order kinetics model and the Langmuir isotherm model accurately described the adsorption process. After five adsorption–degradation cycles, CAs still exhibit a good regeneration ability and stability, which provides valuable insights into the potential application of CAs in efficiently removing, controlling, and remediating aqueous solutions contaminated with 1,4-D.

## Figures and Tables

**Table 1:** Physical properties of 1,4-D.

Property	Value	Structure
Molecular weight	88.11 g/mol	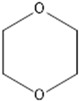
Water solubility	Miscible	
Density (25 °C)	1.0329 g/mL	
Vapor pressure	4 kPa at 20 °C	
Octanol–water partition coefficient (LogK_ow)_	−0.27	
Organic carbon partition coefficient (Log K_oc_)	1.23	
Henry’s law constant at 25 °C	4.8 × 10^−6^ atm m^3^ mol^−1^	

**Table 3:** Pore structure parameters of absorbents.

Sample	S_BET_ (m^2^/g)	S_micro_ (m^2^/g)	S_meso_ (m^2^/g)	V_t_ (cm^3^/g)	V_micro_ (cm^3^/g)	V_meso_ (cm^3^/g)	D (nm)
**CA50-800-1-2** **°**	572	239	333	1.37	0.29	1.08	12.92
**CA100-800-1-2** **°**	638	287	351	1.62	0.29	1.33	9.47
**CA125-800-1-2** **°**	674	275	399	1.81	0.32	1.49	9.70
**CA200-800-1-2** **°**	629	288	341	1.63	0.29	1.34	9.23
**CA500-800-1-2** **°**	406	311	95	0.37	0.16	0.21	8.98
**CA125-800-2-2** **°**	605	258	347	1.54	0.29	1.25	9.11
**CA125-800-1-5** **°**	503	304	199	1.23	0.14	1.08	9.79
**CA125-700-1-2** **°**	452	236	216	1.54	0.12	1.42	13.62
**A560**	480	251	229	0.57	0.14	0.43	4.51
**GAC**	1086	1069	17	0.42	0.39	0.03	3.83

**Table 4:** Pseudo-first- and pseudo-second-order kinetic parameters for 1,4-D adsorption on CA.

C_0_ (mg/L)	Q_e_ (exp) (mg/g)	Pseudo-First-Order Models			Pseudo-Second-Order Models		
		Q_e_ (cal) (mg/g)	k_1_ (min^−1^)	R^2^	Q_e_ (cal) (mg/g)	k_2_ (mg/min)	R^2^
**20**	8.58	5.10	0.0987	0.9904	8.80	0.0478	0.9997
**80**	22.40	10.93	0.0632	0.9778	23.20	0.0122	0.9995
**160**	33.82	11.49	0.0492	0.975	34.72	0.0096	0.9998

**Table 5:** Parameters of Langmuir and Frendlich models for 1,4-D.

T(K)	Langmuir			Freundlich		
	R^2^	Q_m_ (mg/g)	K_L_ (L/mg)	R^2^	1/n	K_F_
**298**	0.97	37.55	0.0138	0.88	0.355	3.818
**308**	0.97	38.69	0.0113	0.86	0.410	2.360
**318**	0.99	67.28	0.0045	0.97	0.546	1.748

**Table 6:** Thermodynamic parameters of adsorption of 1,4-D by CAs.

T (K)	ΔG (kJ/mol)	ΔH (kJ/mol)	ΔS (J/mol/K)
298	−26.83	−52.71	−85.99
308	−26.75		
318	−25.08		

**Table 7:** Comparison of adsorption rates of CA125-800-1-2°, A560 and GAC for the same concentration of 1,4-D at different mass.

Mass (mg)	CA125-800-1-2°	A560	GAC
20	85.81%	75.71%	21.54%
40	91.13%	85.99%	27.45%
60	95.08%	87.89%	33.47%

## Data Availability

Data are contained within the article.
